# Supplementary material for: Longitudinal analysis of blood markers reveals progressive loss of resilience and predicts human lifespan limit
Source: Nat Commun. 2021 May 25;12:2765. doi: 10.1038/s41467-021-23014-1 (PMC8149842; doi:10.1038/s41467-021-23014-1)
Supplement: Supplementary file 1 — Supplementary Information [file 41467_2021_23014_MOESM1_ESM.pdf]

Supplementary Information

Longitudinal analysis of blood markers reveals progressive loss of resilience and predicts human lifespan limit

Timothy V. Pyrkov[a,*], Konstantin Avchaciov[a], Andrei E. Tarkhov[a,b,c],
Leonid I. Menshikov[a,d], Andrei V. Gudkov[e,f] and Peter O. Fedichev[a,c,*]
[a] *Gero PTE, 60 Paya Lebar Road # 05-40B Paya Lebar Square 409051 Singapore*
[b] *Skolkovo Institute of Science and Technology, Bolshoy Boulevard 30, bld. 1, Moscow 121205, Russia*
[c] *Moscow Institute of Physics and Technology, 141700, Institutskii per. 9, Dolgoprudny, Moscow Region, Russia*
[d] *National Research Center "Kurchatov Institute", 1, Akademika Kurchatova pl., Moscow 123182, Russia*
[e] *Roswell Park Comprehensive Cancer Center, Elm and Carlton streets, Buffalo, NY 14263, USA*
[f] *Genome Protection, Inc. 640 Ellicott St #444, Buffalo, NY 14203, USA and*
* *To whom correspondence should be addressed. E-mail: tim.pyrkov@gero.ai, peter.fedichev@gero.ai*

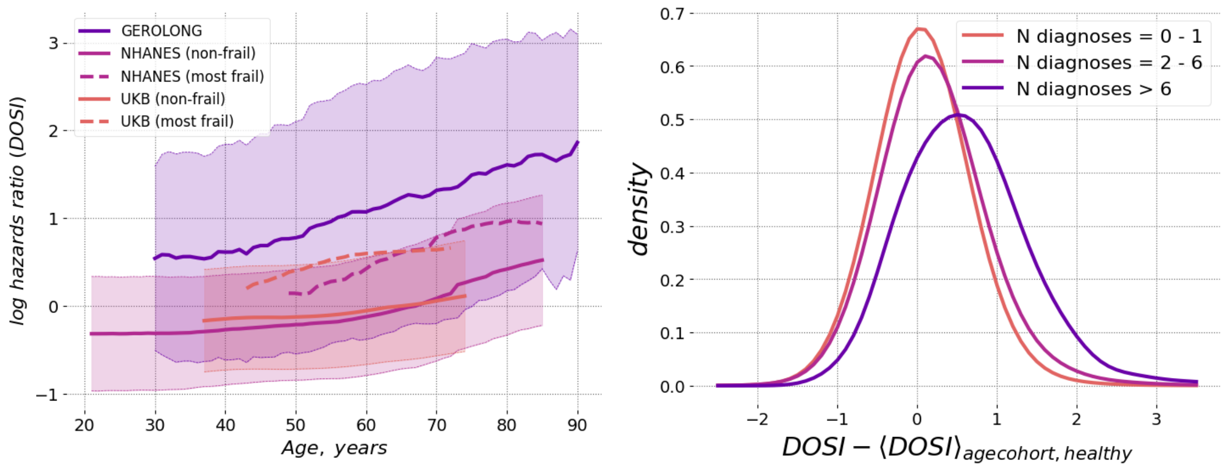

a **b**

Supplementary Figure 1. a. Dynamic organism state indicator (DOSI) mean values (lines) and distribution width (shaded ares representing one standard deviation for each age cohort) are plotted relative to age for the NHANES (same as in Fig. 1B), UKB and GEROLONG datasets (color-matched with respect to each study). For NHANES and UKB the solid line and shaded regions mark the population average ad the range spanned by one standard deviation from it for the "non-frail" ("compound morbidity index", CMI < 0.1) participats. The population mean for the "most frail" (CMI > 0.6) individuals is shown with dashed lines. **b.** Distributions of sex- and age-adjusted DOSI in cohorts of UKB participants in different morbidity categories relative to the DOSI mean in cohorts of "non-frail" (one or no diagnoses, CMI < 0.1) individuals. Note that the distribution function in the "most frail" group (more than 6 diagnoses, CMI > 0.6) exhibited the largest shift and a profound deviation from the symmetric form, similarly as it was seen in NHANES.

Supplementary Table 1: CBC data used in the study.

CBC component	NHANES	UKB
Hemoglobin (g/dL)	LBXHGB	Haemoglobin concentration (30020)
Red blood cell count (million cells/uL)	LBXRBCSI	Red blood cell (erythrocyte) count (20010)
Mean corpuscular volume (fL)	LBXMCVSI	Mean corpuscular volume (30040)
Mean corpuscular hemoglobin concentration (g/dL)	LBXMC	Mean corpuscular haemoglobin concentration (30060)
Red blood cell distribution width (%)	LBXRDW	Red blood cell (erythrocyte) distribution width (30070)
Platelets (1000 cells/uL)	LBXPLTSI	Platelet count (30080)
Neutrophils (%)	LBXNEPCT	Neutrophill percentage (30200)
Lymphocytes (%)	LBXLYPCT	Lymphocyte percentage (30180)
Monocytes (%)	LBXMOPCT	Monocyte percentage (30190)
Eosinophils (%)	LBXEOPCT	Eosinophill percentage (30210)

Supplementary Table 2: Significance of prediction of acquiring a health condition based on estimated log hazards ratio (adjusted for age and gender). Only UKB subjects with none of the listed health conditions at the time of survey were considered; the total number of subjects evaluated for each condition was 266345. The numbers in parentheses in the far right column indicate the occurrence of the disease being the first diagnosis in an individual.

Condition	HR (95% CI)	p-value	n_{events} ($n_{is\ first\ morbidity}$)
Mortality	1.31 (1.29 - 1.34)	1.5e-186	10461 (3628)
First morbidity	1.04 (1.04 - 1.05)	1.3e-29	70562 (70562)
Hypertension	1.03 (1.02 - 1.04)	4.5e-08	32427 (26899)
Arthritis	1.07 (1.05 - 1.08)	1.4e-28	29778 (25339)
Cancers	1.03 (1.02 - 1.05)	5.9e-06	19364 (16284)
Coronary heart disease	1.04 (1.02 - 1.06)	0.00084	7682 (5699)
Diabetes	1.01 (0.99 - 1.03)	0.4	6815 (5453)
Angina pectoris	1.01 (0.98 - 1.04)	0.67	3882 (2278)
Emphysema	1.42 (1.37 - 1.47)	1.1e-83	2467 (1562)
Heart attack	1.04 (0.99 - 1.08)	0.085	2264 (1714)
Stroke	1.09 (1.04 - 1.14)	0.00021	1751 (1239)
Congestive heart failure	1.31 (1.24 - 1.38)	2e-24	1248 (635)
Bronchitis	1.19 (1.07 - 1.32)	0.0014	330 (224)
