# Supplementary material for: Longitudinal analysis of blood markers reveals progressive loss of resilience and predicts human lifespan limit
Source: Nat Commun. 2021 May 25;12:2765. doi: 10.1038/s41467-021-23014-1 (PMC8149842; doi:10.1038/s41467-021-23014-1)
Supplement: Supplementary file 3 — Reporting Summary [file 41467_2021_23014_MOESM3_ESM.pdf]

nature research

Corresponding author(s): Timothy V. Pyrkov

Last updated by author(s): Nov 20, 2020

Reporting Summary

Nature Research wishes to improve the reproducibility of the work that we publish. This form provides structure for consistency and transparency in reporting. For further information on Nature Research policies, see our Editorial Policies and the Editorial Policy Checklist.

Statistics

For all statistical analyses, confirm that the following items are present in the figure legend, table legend, main text, or Methods section.

n/a	Confirmed	
☐	☒	The exact sample size (*n*) for each experimental group/condition, given as a discrete number and unit of measurement
☐	☒	A statement on whether measurements were taken from distinct samples or whether the same sample was measured repeatedly
☐	☒	The statistical test(s) used AND whether they are one- or two-sided<br>*Only common tests should be described solely by name; describe more complex techniques in the Methods section.*
☐	☒	A description of all covariates tested
☒	☐	A description of any assumptions or corrections, such as tests of normality and adjustment for multiple comparisons
☐	☒	A full description of the statistical parameters including central tendency (e.g. means) or other basic estimates (e.g. regression coefficient) AND variation (e.g. standard deviation) or associated estimates of uncertainty (e.g. confidence intervals)
☐	☒	For null hypothesis testing, the test statistic (e.g. *F*, *t*, *r*) with confidence intervals, effect sizes, degrees of freedom and *P* value noted<br>*Give P values as exact values whenever suitable.*
☒	☐	For Bayesian analysis, information on the choice of priors and Markov chain Monte Carlo settings
☒	☐	For hierarchical and complex designs, identification of the appropriate level for tests and full reporting of outcomes
☒	☐	Estimates of effect sizes (e.g. Cohen's *d*, Pearson's *r*), indicating how they were calculated

Our web collection on statistics for biologists contains articles on many of the points above.

Software and code

Policy information about availability of computer code

Data collection	We obtained de-personalized information on: a) CBC measurements from InVitro, the major Russian clinical diagnostics laboratory and b) physical activity records measured by step counts collected by means of a freely available iPhone application.
Data analysis	All data analyses were carried out in python 3.8 scripts using libraries NumPy 1.18.5, SciPy 1.5.2 and Lifelines 0.25.1.

For manuscripts utilizing custom algorithms or software that are central to the research but not yet described in published literature, software must be made available to editors and reviewers. We strongly encourage code deposition in a community repository (e.g. GitHub). See the Nature Research guidelines for submitting code & software for further information.

Data

Policy information about availability of data

All manuscripts must include a data availability statement. This statement should provide the following information, where applicable:

- Accession codes, unique identifiers, or web links for publicly available datasets
- A list of figures that have associated raw data
- A description of any restrictions on data availability

The data that support the findings of this study are available at the NHANES web-site https://www.cdc.gov/nchs/nhanes, at UK Biobank data access procedure described at https://www.ukbiobank.ac.uk/enable-your-research. Additional data are available from the corresponding author on reasonable request.

Field-specific reporting

Please select the one below that is the best fit for your research. If you are not sure, read the appropriate sections before making your selection.

- [x] Life sciences
- [ ] Behavioural & social sciences
- [ ] Ecological, evolutionary & environmental sciences

For a reference copy of the document with all sections, see nature.com/documents/nr-reporting-summary-flat.pdf

Life sciences study design

All studies must disclose on these points even when the disclosure is negative.

Sample size	We used cross-sectional Complete blood counts (CBC) measurements from UK Biobank (471473 subjects) and NHANES (72925 subjects) datasets. We used longitudinal data for CBC (1082 subjects) and for step counts (4857 subjects). The number of datasets used in the study was determined based on the goals of our work: a) test concordance of results between different populations enrolled in UK Biobank and NHANES, b) study fluctuations in longitudinal blood-based marker and physical activity measurements.
Data exclusions	The fraction of samples with missing (or filled with zero) CBC data was less than 0.035% in any studied dataset and those samples were discarded.
Replication	We performed retrospective analyses and therefore no data replication was used. Results were validated using cross-validation procedure where necessary in response to reviewers' comments and reported in the updated version of the manuscript.
Randomization	NHANES population aged 40-85 y.o. was split randomly into training (12851 participants) and test (12883 participants) subsets.
Blinding	We performed retrospective analyses and therefore blinding was not applied. Results were validated using cross-validation procedure where necessary in response to reviewers' comments and reported in the updated version of the manuscript.

Reporting for specific materials, systems and methods

We require information from authors about some types of materials, experimental systems and methods used in many studies. Here, indicate whether each material, system or method listed is relevant to your study. If you are not sure if a list item applies to your research, read the appropriate section before selecting a response.

Materials & experimental systems

n/a	Involved in the study	
x		Antibodies
x		Eukaryotic cell lines
x		Palaeontology and archaeology
x		Animals and other organisms
x		Human research participants
x		Clinical data
x		Dual use research of concern

Methods

n/a	Involved in the study	
x		ChIP-seq
x		Flow cytometry
x		MRI-based neuroimaging
